# Handshake stewardship on adult acute-care surgical services

**DOI:** 10.1017/ash.2023.267

**Published:** 2023-09-29

**Authors:** Elizabeth Neuner, Kevin Hsueh, Michael Durkin, Sena Sayood

## Abstract

**Background:** Handshake stewardship is a variation of prospective audit and feedback that entails the individual review of patient charts by a physician–pharmacist collaborative team followed by in-person feedback to primary teams to communicate recommendations regarding optimal antibiotic use. Handshake stewardship has been shown to have durable effects in reducing antimicrobial use in children’s hospitals, but data regarding this intervention in adult hospitals are scarce. In particular, no data are available regarding the impact of this type of stewardship intervention on adult surgical units. We examined the effect of a handshake stewardship intervention at a large academic medical center on adult trauma and acute- and critical-care surgery (ACCS) units. **Methods:** The antimicrobial stewardship program (ASP) at Barnes-Jewish Hospital launched a handshake stewardship intervention targeting surgical floor teams in January 2022. These teams included the ACCS teams and a number of other surgical services. The intervention consisted of once weekly reviews and in-person rounds with the surgical floor teams along with the establishment of a 7 day per week “hotline” in which the surgical teams could contact an ID physician or pharmacist with questions regarding antibiotic use. Patients with formal ID consultations were not reviewed. Recommendations were tracked including the type, the antibiotic targeted, and recommendation acceptance or rejection. Descriptive statistics were performed to analyze these results. At the end of 12 months, antibiotic use in the floors covered by the ACCS teams were pulled from the NHSN AU module to perform an interrupted time-series analysis 12 months before and after the intervention. **Results:** Overall, 3,127 charts were reviewed during the intervention period and 637 recommendations were made to all the surgical teams. Opportunities for antibiotic use optimization were identified in ~20% of antibiotic orders. The overall recommendation acceptance rate was 71%. In the ACCS units, 272 interventions were recommended, with an acceptance rate of 67%. The most frequent recommendations were for antibiotic discontinuation (37%), antibiotic de-escalation (17%), shortening duration (12%), and broadening coverage (12%). Antibiotic usage trends (Fig. 1) on the ACCS floors, which were showing a nonsignificant increasing trend (*P* = .70) before and after the intervention, now show a nonsignificant decreasing trend (*P* = .20). **Conclusions:** There are numerous opportunities for antibiotic optimization on adult surgical floors. Although handshake stewardship is a labor-intensive intervention, preliminary findings after 1 year show that, on ACCS units, there may be a trend toward a sustained impact.

**Figure:**
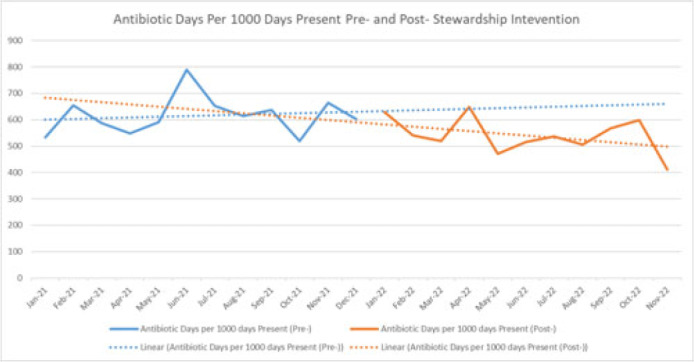

**Figure:**
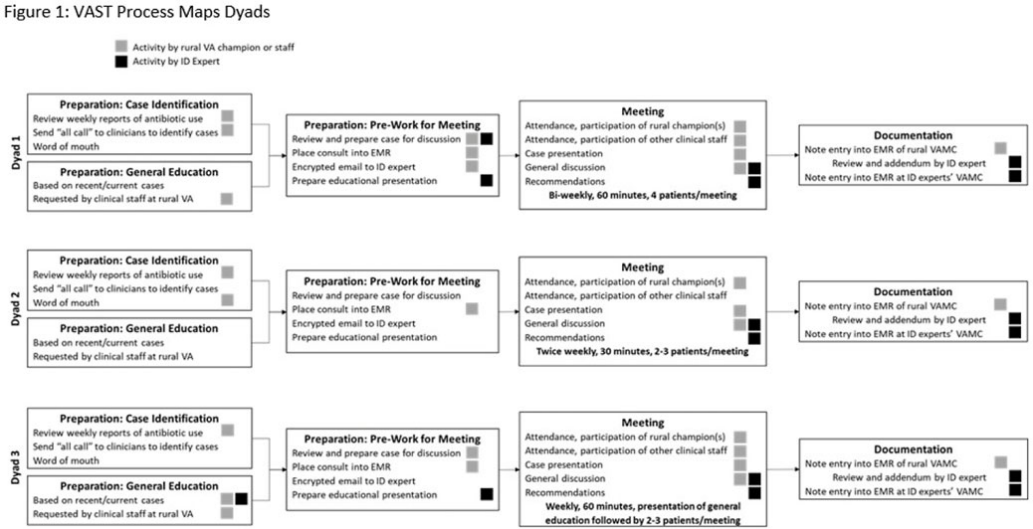

**Disclosures:** None

